# Changes in the Substrate Source Reveal Novel Interactions in the Sediment-Derived Methanogenic Microbial Community

**DOI:** 10.3390/ijms20184415

**Published:** 2019-09-08

**Authors:** Anna Szafranek-Nakonieczna, Anna Pytlak, Jarosław Grządziel, Adam Kubaczyński, Artur Banach, Andrzej Górski, Weronika Goraj, Agnieszka Kuźniar, Anna Gałązka, Zofia Stępniewska

**Affiliations:** 1 Department of Biochemistry and Environmental Chemistry, Institute of Biotechnology, The John Paul II Catholic University of Lublin, Konstantynów Street 1 I, 20-708 Lublin, Poland; 2 Department of Agricultural Microbiology, Institute of Soil Science and Plant Cultivation–State Research Institute (IUNG-PIB), Czartoryskich Street 8, 24-100 Puławy, Poland; 3 Institute of Agrophysics, Polish Academy of Sciences, Doświadczalna Street 4, 20-290 Lublin, Poland

**Keywords:** methanogenesis, bottom sediments, enrichment culture, *Caldiserica*, *Methanothrix*, *Methanomassiliicoccus*

## Abstract

Methanogenesis occurs in many natural environments and is used in biotechnology for biogas production. The efficiency of methane production depends on the microbiome structure that determines interspecies electron transfer. In this research, the microbial community retrieved from mining subsidence reservoir sediment was used to establish enrichment cultures on media containing different carbon sources (tryptone, yeast extract, acetate, CO_2_/H_2_). The microbiome composition and methane production rate of the cultures were screened as a function of the substrate and transition stage. The relationships between the microorganisms involved in methane formation were the major focus of this study. Methanogenic consortia were identified by next generation sequencing (NGS) and functional genes connected with organic matter transformation were predicted using the PICRUSt approach and annotated in the KEGG. The methane production rate (exceeding 12.8 mg CH_4_ L^−1^ d^−1^) was highest in the culture grown with tryptone, yeast extract, and CO_2_/H_2._ The analysis of communities that developed on various carbon sources casts new light on the ecophysiology of the recently described bacterial phylum *Caldiserica* and methanogenic *Archaea* representing the genera *Methanomassiliicoccus* and *Methanothrix*. Furthermore, it is hypothesized that representatives of *Caldiserica* may support hydrogenotrophic methanogenesis.

## 1. Introduction

Microbial diversity and functioning in the environment are one of the most intriguing issues in science, given that they are related to the foundations of the knowledge regarding biogeochemical cycles. Recent methodical advances, in particular the introduction of next generation sequencing, facilitate the description of microbiomes with unprecedented depth [1,2]. It is very important to recognize microbial communities in the natural environment in terms of their composition and function, as achievements in this field not only contribute to overall knowledge but also provide a basis for biotechnological progress [3,4]. One of the most common objects of this type of research are anoxic environments, which are a source of microbiota that can be employed in biogas production due to their physiological capabilities [5,6]. Methanogenesis is a multistage process in which degradation of complex organic molecules into simple one-carbon compounds is performed by a consortium of microorganisms [7,8]. Their existence in the natural environment is an effect of coevolution leading to the development of metabolic interactions that allow the flow of carbon, energy, and other intermediates for mutual benefit [9,10]. The best recognized methanogenic *Archaea* belong to the phylum *Euryarchaeota* and are classified into seven orders, namely *Methanococcales*, *Methanobacteriales*, *Methanosarcinales*, *Methanomicrobiales*, *Methanopyrales*, *Methanocellales*, and *Methanomassiliicoccales* [11]; yet, additional new phyla, namely *Bathyarchaeota* [12] and *Verstraetearchaeota* [13], have been postulated. Methanogens use a narrow range of substrates such as H_2_/CO_2_, formate, acetate, methanol, methylated compounds, and CO. Ethanol and 2-propanol may be used by some methanogens instead of H_2_ as electron donors [11]. This narrow range of substrates is delivered by bacteria, mostly from the genera *Clostridium, Pseudomonas, Desulfovibrio,* and *Cellulomonas* [14]. Organic matter, in freshwater lakes, is primarily decomposed by bacteria to acetate, CO_2_, and H_2_, which are the main substrates used by methanogens in two major pathways: hydrogenotrophic and acetotrophic [15,16].

The biotechnological potential of anaerobic microbial communities is closely connected with their biodiversity [17]. The highest biodiversity has been found in transitional zones called ecotones [18]. The observations that were first made on the macroscale (for higher plants and animals) [19] revealed the highest biodiversity at the boundaries of forests and meadows [20], or lakes and terrestrial ecosystems [21], and have been confirmed on the microscale as well [22]. Microbial biodiversity (in terms of the structure and function) in zones located between forests and grasslands [22], lakes and rivers [23,24], was found to be higher than in adjacent ecosystems. Such locations may thus have a hidden potential for biotechnology. The present study was focused on a microbial community found in the sediment of a shallow reservoir, Szczecin, that has developed over the last three decades in subsidence resulting from mining activity. Due to the continuous exploitation of coal, the area and depth of the reservoir are increasing continuously. It is also exposed to huge temperature and insolation fluctuations resulting from the climatic conditions of the mid latitudes. Consequently, the sediment is subjected to continuous changes in temperature and aeration. Furthermore, the submergence of subsequent fragments of agricultural soils leads to the release of huge amounts of biogenic compounds, and an additional portion thereof is transported from adjacent fields by surface runoff. The imbalance in the ecosystem is manifested by algal blooms occurring in the reservoir each summer. Oxygen deficiencies and organic matter supply from decaying algae create conditions that support biogenic methane (CH_4_) formation, which is a result of the cooperation of methanogens with fermentative microorganisms.

Generally, the biodiversity and metabolic activities of bacterial communities in lake sediments decrease with the depth in the profile [25]. Hence, the surface layer of the bottom sediments of the Szczecin reservoir was chosen to study microbial ecology and a possible source of microorganisms that can potentially be used in biogas production.

Sediment-derived enrichment cultures grown on various carbon substrates were used as a tool to elucidate the relationship between microbial community composition and methane production rate. Cultivation largely reduces the initial biodiversity. On the other hand, targeted selection of culture medium components may lead to the enrichment of microorganisms exhibiting the desired abilities and provide new insight into their ecophysiology. The complexity and multistage nature of the biological CH_4_ formation process suggest that investigations are only valid when the community structure is studied using high throughput methods. 

The specific aim of this study was to determine the link between the microbiome composition and methane production and to describe changes occurring in the methanogenic community structure and its metabolic capabilities as a function of the enrichment stage and medium composition.

## 2. Results

The investigated sediments were characterized by slightly acidic reaction (5.46), redox potential (Eh) below −265 mV, and organic carbon content equal to 48 g kg d.w.^−1^. The content of N-NH_4_ forms was almost three times higher than the concentration of bioavailable phosphorus (P-PO_4_) (Table 1).

### 2.1. Methane Production

**Figure 1:**
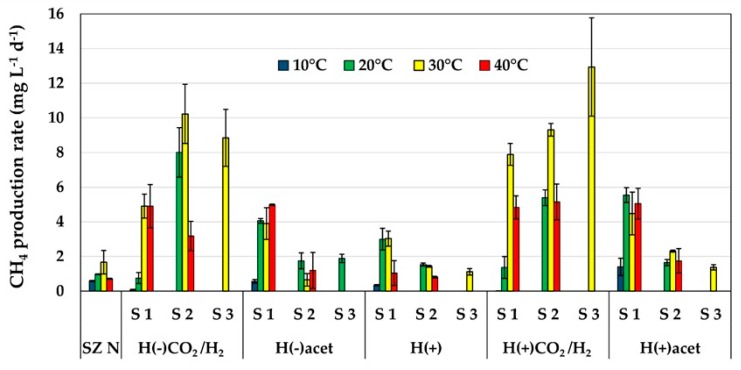
Methane production rate in particular media and stages of enrichment. Mean values with standard deviation (SD) are presented.

CH_4_ production in the sediment samples (tested in laboratory conditions at temperatures 10–40 °C) revealed a maximum of over 1.67 mg CH_4_ L^−1^ d^−1^, at 30 °C. The enrichment cultures were prepared in the same temperature range and in mineral media supplemented with different carbon sources. In the medium H(+), the highest methane production rate (MP, above 2.99 mg CH_4_ L^−1^ d^−1^, at 20 and 30 °C) was determined during stage S1. In the subsequent stages S2 (20, 30 °C) and S3 (30 °C), the activities decreased and were significantly lower, ca. 1.28 (20, 30 °C) and 1.12 mg CH_4_ L^−1^ d^−1^ (30 °C), respectively. The supplementation of the medium with sodium acetate (H(+)acet) resulted in MP of approximately 5.54 mg CH_4_ L^−1^ d^−1^ in the S1 stage, whereas one third of that value was noted in S2 and S3. Substantially higher MP was revealed when the headspace gas in the serum vials was replaced by methanogenic substrates: CO_2_ and H_2_ (20:80 *v*/*v*). In this case, the optimal temperature was 30 °C. MP increased with each subsequent stage of incubation. It was the highest in S3 (exceeding 12.8 mg CH_4_ L^−1^ d^−1^). This value was also the highest among all the experimental treatments. When the medium was free of tryptone and yeast extract but supplemented with sodium acetate (H(−)acet), methane production above 3.8 mg CH_4_ L^−1^ d^−1^ was observed at 20–40 °C in S1. In S2, MP decreased significantly, maximally by 83% with optimal thermal conditions noted at 20 °C. At this temperature, the CH_4_ production rate reached 1.74 mg CH_4_ L^−1^ d^−1^, and the reduction of MP was the lowest but still substantial, i.e., 57%, compared to S1. In S3, the value of MP was similar to that in S2. In the H(−) medium and the CO_2_ and H_2_ atmosphere (20:80 *v*/*v*), MP was the highest at 30 and 40 °C in S1. However, in the next stage (S2), 30 °C appeared to be more suitable for methanogenesis, with MP reaching as much as 10.23 mg CH_4_ L^−1^ d^−1^. In S3, MP was still high, i.e., 8.85 mg CH_4_ L^−1^ d^−1^ (Figure 1).

The changes in the duration of the lag phase of MP indicate that the microbial consortia that developed in all of the treatments are able to metabolize the substrates added and create suitable conditions for methanogenesis. The lag phase was the longest in the initial phase of the experiment (S1) and shortened after the subsequent transfers (Table 2). The rise in temperature also caused a reduction in the lag phase. Starting from S2, in almost all experimental treatments, at 30 and 40 °C, methane production began within one day of inoculation. The only exception was the community grown with acetate as the sole carbon source (H(−)acet). This community was not only characterized by the longest lag time but also saw optimal growth at 20 °C.

### 2.2. Physicochemical Conditions

The pH value at the beginning of the incubation in S1 was generally slightly higher than at the end (Table 3). In the subsequent transitions, changes in the reaction depended on the treatment applied. Generally, H_2_ and CO_2_ resulted in slight acidification of the medium, while addition of organic carbon substrates promoted alkalization (Table 3). Based on the oxidation-reduction potential, it was found that the microbial communities were able to reduce the growth medium, with efficiency increasing from S1 to S3. 

In the first stage of the experiment (S1), optical density (OD_600_) was not measured due to the addition of a source material containing insoluble particles. In stages 2 (S2) and 3 (S3), the increase in turbidity was confirmed in all treatments, and significantly larger changes were revealed in the combinations with medium supplemented with yeast extract and tryptone (H+) and acetate addition than in those containing solely acetate or CO_2_/H_2_ (Table 3). 

### 2.3. Microbial Community Structure Across Sediment and Media Combinations

**Figure 2:**
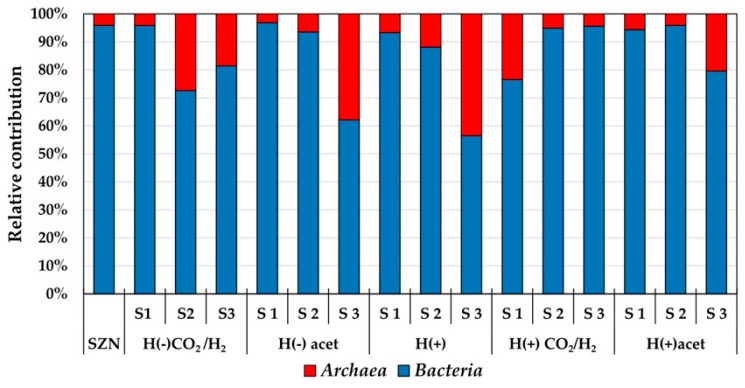
*Archaea* and *Bacteria* contribution in the particular stages of the experiment.

As shown by the 16S rRNA gene analysis, *Bacteria* was the predominant domain in the sediment and enrichments in all stages of the experiment (Figure 2). The relative contribution of *Archaea* tended to increase with the successive stages of culture in media H(−)acet, H(+), and H(+)acet. *Archaea* species were the most prominent in S3 of the incubation in media H(+) and H(−)acet and accounted for 43 and 38%, respectively, of the total microbial community. In media H(−)CO_2_/H_2_ and H(+)CO_2_/H_2_, the *Archaea* contribution decreased in the subsequent stages (Figure 2). The *Archaea* domain was dominated by methanogens, which accounted for almost 99% of the total *Archaea*. 

**Figure 3:**
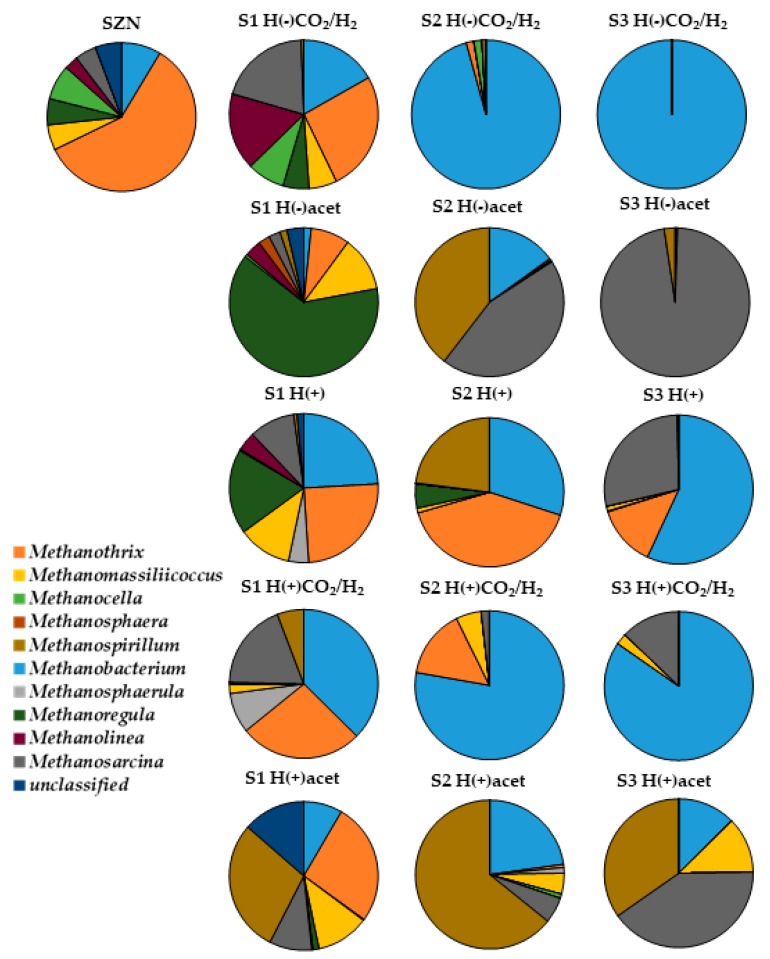
Changes in the methanogen community during incubation in different media.

Representatives of the genus *Methanothrix* were the dominant methanogens in the bottom sediment; additionally, *Methanocella*, *Methanobacterium*, *Methanoregula*, *Methanomassiliicoccus*, *Methanosarcina*, and *Methanolinea* species and some methanogens that were not classified to any genus were identified (Figure 3). The culture in each medium resulted in differentiation of the methanogen composition. In the last step of the culture (S3), methanogenic communities grown on media H(−)CO_2_/H_2,_ H(+), and H(+)CO_2_/H_2_ were dominated by *Methanobacterium*, which accounted for 100%, 57%, and 85% of the identified methanogens, respectively. In media H(−)acet and H(+)acet, the main methanogen represented *Methanosarcina* (97% and 41% of all the methanogens, respectively). The second important genus identified in consortia growing in H(+)acet was *Methanospirillum* (35%). The consortium from the medium combination H(+) additionally contained species from *Methanothrix* (13%), while *Methanomassiliicoccus* identified in H(+)acet and H(+)CO_2_/H_2_ accounted for 12% and 2% of the cultured methanogens, respectively.

The presence of non-methanogenic *Archaea* (*Crenarchaeota* (1.55%) *Woesearchaeota* (0.55%), *Pacearchaeota* (0.26%) *Diapherotrites* (0.015%), and *Thaumarchaeota* (0.015%)) was confirmed only in the native bottom sediment.

**Figure 4:**
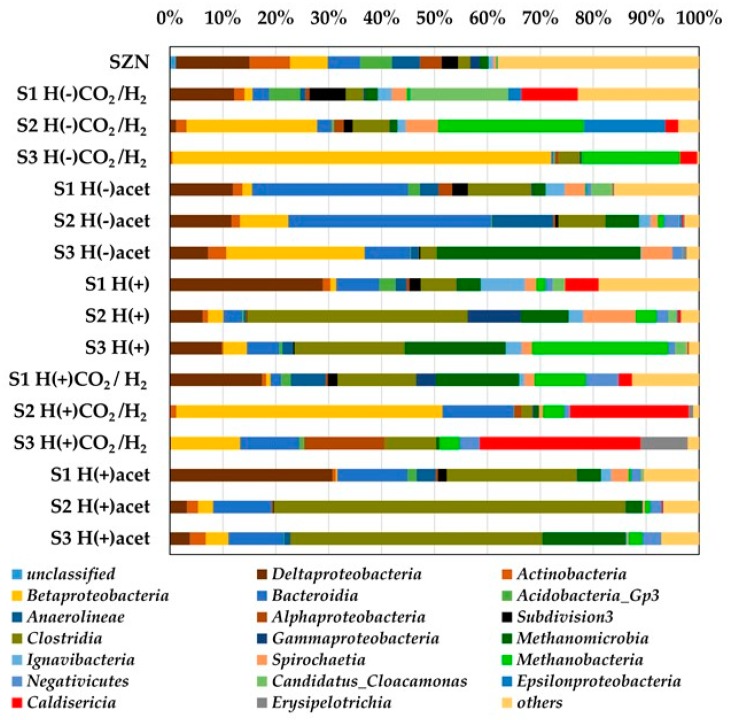
Diversity in the microbial communities at the class level (microbial classes represented by >2% of total sequences, classes making up less than 2% were classified as others) in response to the different media composition and in the particular stage of enrichment identified by 16S rRNA gene sequences.

The structure of the microbial communities was clearly differentiated by available carbon sources. At the class level (Figure 4), *Deltaproteobacteria* (above 12%) dominated in the sediment (SZN) followed by *Actinobacteria* (6.2%), *Betaproteobacteria* (5.3%), *Bacteroidia* (5.3%), and *Acidobacteria* (5.3%), which had smaller but significant contribution in the consortium. In the first stage (S1), *Deltaproteobacteria* dominated in H(+)acet, H(+)CO_2_/H_2_, and H(+), *Candidatus Cloacamonas* in H(−)CO_2_/H_2_, and *Bacteroidia* in H(−)acet. The proportion of the identified microorganisms changed during the next stages of the culture. In medium H(−)CO_2_/H_2_ in the subsequent stages, an increasing proportion of *Betaproteobacteria* was noted (from 1.25% at the beginning to over 71% in the last stage of the culture). Increased contribution was also found in the case of *Methanobacteria* (from 0.4% to 26% and 18% in stages 2 and 3, respectively). The contribution of *Betaproteobacteria* increased with successive stages of the experiment when acetate was used (H(−)acet, from 1.6 to 25.6%) as a sole carbon source, as in the case of *Methanomicrobia*, (from 2.1% to 37.7%). An increase in the contribution of *Methanomicrobia* was also noted in the subsequent stages of H(+) media enrichment (from 3.8% to 18.2%). In the medium supplemented with multiple carbon sources H(+)CO_2_/H_2_ (tryptone, yeast extract, and CO_2_), there was a significant increase in the contribution of *Caldisericum* (to over 30%) between the first and last stages. Media based on tryptone, yeast extract, and acetate (H(+)acet) caused an increase in the contribution of *Clostridia* as well as *Methanomicrobia* in the microbial community structure (Figure 4). 

### 2.4. Microbial Biodiversity 

The microbial taxonomic diversity in the SZN sediment was high, which was reflected in the values of the biodiversity indices (Table 4). Analysis of the enrichment cultures revealed that the composition of the medium had a great impact on biodiversity. All indices used to describe the community composition as a function of the substrates added and the transition stage have shown that the H(−)CO_2_/H_2_ and H(−)acet treatments in the first cultivation stage were characterized by the greatest biodiversity and harbored nearly half of the OTUs found in SZN. A great majority of OTUs found in the initial stages of the H(−)CO_2_/H_2_ and H(−)acet treatments was lost during the subsequent transitions. The decrease was especially visible in the community that developed in H(−)CO_2_/H_2_, where only 96 OTUs were detected in the final stage of the experiment. Surprisingly, similarly low final biodiversity (97 OTUs) was found in S3 of the H(+)CO_2_/H_2_ variant. These two treatments also revealed the highest methanogenic activity which increased with subsequent transitions. The selection is confirmed by the values of the inverse Simpson index, which is strongly affected by the evenness of abundant species. Communities that developed in H(+)CO_2_/H_2_ and H(−)CO_2_/H_2_ in S3 were characterized by very low values of this index (7.6 and 2.1, respectively), which confirms that they were dominated by only a few microbial taxa (Table 4). The microbial consortia found in the other treatments were much more resistant to biodiversity loss. Interestingly, nearly 240 OTUs were still present in the communities grown on H(+) and H(+)acet in the final stage of cultivation (S3). 

**Figure 5:**
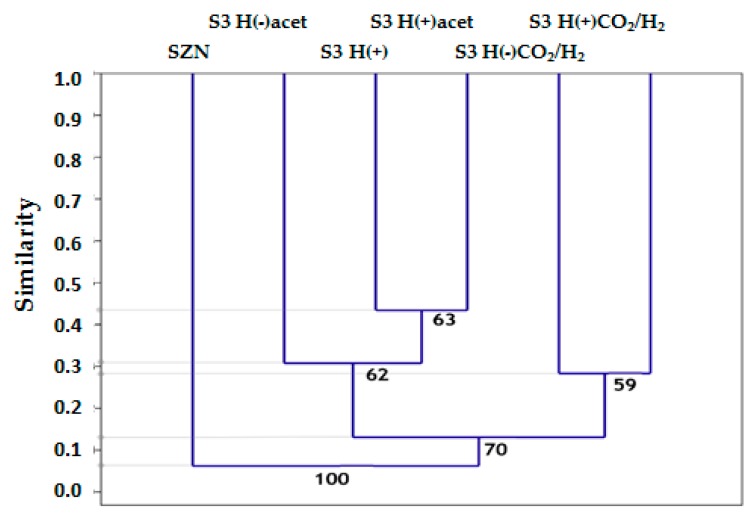
Beta-diversity of the bacterial communities in the experimental treatments. The similarity presented on the scale was calculated based on the Bray-Curtis distances, and the tree was generated by an UPGMA algorithm. The bootstrap number was set to 999.

The beta-diversity analysis confirmed the significance of differences between the communities. The reconstructed tree describing divergence has shown clearly that the substrates added to the culturing medium were the main factors affecting the composition of the SZN-derived communities (Figure 5). CO_2_/H_2_ seems to be the primary differentiating factor_._ Communities that developed in H(+)CO_2_/H_2_ and H(−)CO_2_/H_2_ formed a separate branch and exhibited only a 15% similarity to those grown on solely organic compounds. The differences between the communities grown on organic substrates were smaller.

## 3. Discussion

### 3.1. Lake Sediment

Methane production in the sediment (SZN) ranged from 0.58 to over 1.67 mg CH_4_ L^−1^ d^−1^ depending on the incubation temperature and with a maximum at 30 °C. These values were in the range of the methane production determined for river and pond sediments investigated by Yang [26] in northern Taiwan (0.24–18.0 mg CH_4_ L^−1^ d^−1^) and for Lake Kinneret in Israel (0.38–7.68 mg CH_4_ L^−1^ d^−1^) investigated by Schwarz and co-workers [27]. Methane formation was also higher than that found in sludges from acidic bog lakes Grosse Fuchskuhle (Germany) (0.032–0.368 mg CH_4_ L^−1^ d^−1^) [28]. 

Methanogens, with the dominant *Methanosarcinales* (*Methanothrix* and *Methanosarcina*) able to utilize a number of different substrates, e.g., CO_2_, H_2_, and acetate (Figure 3), were responsible for the methane production in the Szczecin reservoir sediment. The contribution of representatives of *Methanobacteriales*, *Methanomicrobiales*, *Methanocellales* and *Methanomassiliicoccales* was much lower. This situation is different from that in eutrophic and mesotrophic lakes (Dianchi and Erhai, China) where the dominance of *Methanomicrobiales* and *Methanobacteriales* was confirmed, and, despite the slightly acidic reaction of the Szczecin reservoir sediment, was similar to the alkaline soda lake (Mono Lake, CA, USA) dominated by *Methanosarcinales* and *Methanomicrobiales* [29,30]. A similar methanogenic community structure was also found in several Amazonian lake sediments with variable pH (5.7 to 8.1) [31]. *Deltaproteobacteria*, *Actinobacteria*, *Betaproteobacteria*, *Bacteroidia*, and *Acidobacteria* (more than 5% contribution) dominated among the accompanying bacteria in the Szczecin reservoir sediment. The microbial community was slightly similar to that detected in eutrophic Chinese lakes, where *Deltaproteobacteria*, *Betaproteobacteria*, *Gammaproteobacteria*, *Alphaproteobacteria*, *Anaerolineae*, and *Nitrospira* were the dominant classes [32].

### 3.2. Enrichment Cultures 

In each treatment, MP was observed after the lag phase. Its occurrence at the beginning of the culture-based experiment results from adaptation to changed conditions [33]. In our experiment, where the CH_4_ dynamics was of central importance, the lag phase duration reflects the time necessary for reduction of the culture medium to sufficiently low redox conditions. It could be expected that biodiversity loss would impair self-reduction potential of the microbial community. Our experiment shows that sediment derived from an instable subsidence reservoir (Szczecin) harbors a community that is able to maintain (or even increase) the self-reduction potential in spite of the substantial reduction of biodiversity. Furthermore, although the lag phase was the shortest in the cultures grown with H_2_ (which is a reducing agent itself) other substrates also supported the fast creation of conditions suitable for methanogenesis, which is important when considering further research of the biotechnological potential of the community.

### 3.3. Microbial Biodiversity

The biodiversity in the SZN sediment (Table 4) was similar to that in other lake sediments worldwide (analyzed using the analogous sequencing depth) [34,35] and higher than values typically calculated for soil [36,37] or freshwater [38,39]. This confirms that the transitional environments of the sediments harbor much higher microbial diversity that the adjacent environments. 

Microbial diversity was largely lost in the enrichments. The highest decline was noted between SZN and S1 of the cultures for all calculated indexes (Table 4). It should be noted that the trophic structure of the microbial community in the S1 cultures was still influenced by the availability of organic matter originating from the basal sediment (dilution 2 × 10^−2^). The second stage S2 was transitional, while at the third stage of cultivation when the availability of the basal material was 8 × 10^−6^, the community was in fact dependent only on substrates delivered by the experimental medium. The decrease in biodiversity between the second and third stage of culturing was low, which suggests that the S3 community structures were determined mainly by the experimental conditions. 

Subsequent transitions resulted in a decrease in unidentified sequences, which probably points to the disappearance of species that are not able to grow when deprived of the natural sediment and are unculturable to date. Furthermore, it was found that a majority of the genera that were lost during the successive stages of the experiment were aerobic (*Gaiella*, *Gemmatimonas, Nitrospira*, *Conexibacter, Methylobacter, Thermoleophilum*, *Kofleria)* or microaerobic *(Sideroxydans*, *Magnetococcus*, *Anaeromyxobacter*, and *Sterolibacterium*—threshold of 1% of the reads in SZN. The presence of aerobic microorganisms confirms the transitional character of the SZN sediment and the dualistic character of the microbial community (aerobic/anaerobic). The loss of aerobes was a consequence of the application of the culturing conditions.

The structure of the microbial communities was shaped by the available carbon sources (Figure 4). Adaptation to the culturing conditions (available carbon sources) was also clearly visible for the methanogens. *Methanothrix*, which is the most frequent *Archaea* in the SZN sediment, disappeared gradually in a majority of the treatments. In the third stage of the experiment, it was outcompeted by other methanogenic genera (Figure 3). The differentiation reflected the metabolic capabilities of *Archaea*. The availability of the thermodynamically beneficial energy source (H_2_) led to increased contribution of hydrogenotrophic *Methanobacterium* (in H(+), H(−)CO_2_/H_2_, and H(+)CO_2_/H_2_) while the addition of acetate to the culture medium (H(+)acet and H(−)acet promoted *Methanosarcina*. In this case, our experiment confirms the previously described fact that although both are acetotrophic, *Methanothrix* and *Methanosarcina* differ in the kinetics of enzymes involved in acetate assimilation. Hence, low acetate concentrations favour *Methanothrix*, which is outcompeted by *Methanosarcina* at high acetate levels [40].

*Methanothrix* almost completely disappeared at S3 of the experiment. Only in the H(+) culture, at S3, did it still account for 13% of the methanogens (Figure 3). If the outcompetition of *Methanothrix* by *Methanosarcina* can indeed be explained by competition for the substrate, the disappearance of *Methanothrix* in H(+)acet and H(−)acet requires additional explanation. The composition of the basal medium (vitamins and trace element content) was the same in all cultures. Therefore, it seems that the primary cause of the changes in methanogen contribution would be the associated bacterial community (differentiated by the available carbon source). 

*Methanothrix* may lack some key compounds, e.g., auxotrophic vitamins or amino acids. Recently, Hubalek et al. [41] presented a comprehensive study showing that the proportion of genes encoding auxotrophy for vitamins and amino acids in the metagenome-assembled genomes of anaerobic, hydrocarbon-degrading communities is surprisingly high compared to those linked with energy conservation. This led them to the conclusion that metabolic interactions between obligate mutualistic microbial partners should be of central importance because beyond the canonical H_2_-producing and syntrophic bacteria - methanogen partnership, a complex (although not fully defined) interactions play an important role in determination of the metabolism of the entire community.

Our study shows that the cooperation between acetotrophic methanogens (*Methanothrix*) and acetogenic bacteria may be one of those relationships. It has already been proven that *Methanosarcina* owe its physiological flexibility to *Clostridia*—the most probable source of a unique (as for *Archaea*) enzymatic system employing acetate kinase (AckA) and phosphoacetyl transferase (Pta) [42]. Enzymes involved in methane production from acetate may not be the only ones “imported” via gene transfer. It cannot be excluded that other genes, not yet identified but enhancing survival in environmental conditions, were also incorporated by *Methanosarcina*. *Methanothrix* seems to lack such benefits. In this work, we found a relationship between the contribution of *Methanothrix* and acetogenic bacteria (the latter presented as KEGG-revealed expected abundances of genes responsible for the synthesis of enzymes involved in the acetate-generating Wood–Ljungdahl pathway - carbon monoxide dehydrogenase [EC 1.2.7.4]/acetyl-CoA synthase [EC 2.3.1.169]). We hypothesize that *Methanothrix* gains more benefits from cooperation with acetogenic bacteria than from substrate delivery and interspecies electron transfer. Examples of such a relationship have already been demonstrated in co-cultures (alanine transfer between *Methanococcus maripaludis* and *Desulfovibrio vulgaris* [43]). 

**Figure 6:**
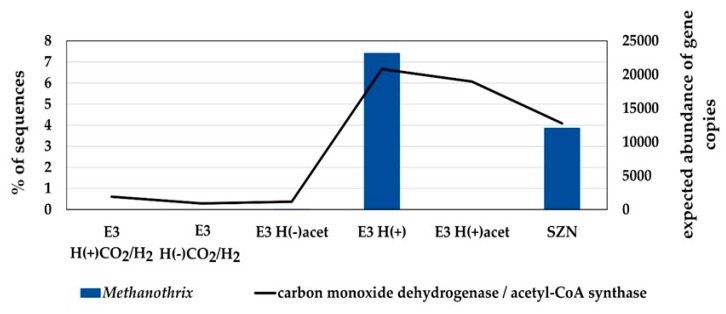
Relative contribution of *Methanothrix* and acetogenic bacteria (the latter presented as KEGG-revealed expected abundances of genes responsible for the synthesis of enzymes involved in the acetate-generating Wood–Ljungdahl pathway - carbon monoxide dehydrogenase [EC 1.2.7.4]/acetyl-CoA synthase [EC 2.3.1.169].

A support for the deduced relationships seems to be the disappearance of *Methanothrix* in treatments containing acetate, where the growth of acetogenic bacteria was inhibited by excess product concentration (*via* a mechanism described previously by Wang and Wang [44] (Figure 6). Full confirmation of the necessity of *Methanothrix* – acetogen cooperation requires comprehensive studies. The use of model co-cultures subjected to transcriptional, proteomic and metabolic analyses or shotgun metagenome/metatranscriptome analyses of environmental samples would explain the exact nature of the deduced cooperation between *Methanothrix* and acetogenic bacteria. 

Interestingly, the proportion of methanogens was not in line with the methane production rate detected in vivo (Figure 1). The treatment that exhibited the highest methane production (growing with each transfer and characterized by the shortest lag phase) was also characterized by a very low proportion of the identified methanogens (4.3% of the reads). Instead, unexpectedly, high contribution of *Caldiserica* was found in H(+)CO_2_/H_2_. These bacteria are one of the most intriguing elements of the SZN consortium. *Caldiserica*, formerly known as OP5, was first described based on environmental 16S rRNA fragments isolated from Obsidian Pool (Yellowstone) [45]. The first culturable species of the phylum, i.e., *Caldisericum exile*, was isolated by Mori and co-workers a decade later [46] from a hot spring in Japan. In the present experiment, *Caldiserica* was found in almost all of the treatments and was particularly abundant in H(+)CO_2_/H_2_. The culturing conditions in which CO_2_/H_2_, yeast extract, and tryptone were used were highly suitable for these bacteria, whose participation in the community structure grew successfully with the consecutive culture stages reaching over 30% of the sequence reads in S3. In the other variants containing H_2_/CO_2_, yeast extract, and tryptone separately, they accounted for 0.17% and 3.2% of the sequences in the final stage of the experiment. Almost no *Caldiserica* representatives were detected in the treatments containing acetate (even in the presence of yeast extract (H(+)acet)). 

*C. exile*, the only known culturable representative of the phylum to date, was described as anaerobic, thermophilic, and thiosulfate-reducing bacterium. Therefore, it could be expected that the presence of *Caldiserica* would hinder methane production by competition for hydrogen with methanogens. In this work, we found that the presence of huge numbers of *Caldiserica* did not reduce methanogenesis but seemed to even stimulate it. Similar observations have been reported by Ma and co-workers [47], who investigated degradation of hexadecane to methane as a function of sulphate concentration. In that research, the most effective culture (containing 0.5 mM sulphate) contained a high proportion of *Caldiserica*. These authors did not emphasize the role of *Caldiserica* but their analysis of the whole community suggested that there is a possibility of cooperation between incomplete-oxidizing sulphate reducers and methanogens, as incomplete oxidation of organic intermediates may generate H_2_ through sulphate reduction. In fact, sulphur disproportionation may be carried out with protons being either a substrate or a product of the reaction, as described below (Equations (1), (2)) [48,49]:

S_2_O_3_^2−^ + 2H^+^ + 2e^−^→ HS^−^ + HSO_3_^−^ (1)

S^2^O_3_^2−^+H_2_O → SO_4_^2−^+HS^−^+H^+^ (2)

The ecology of *Caldiserica* is currently being discovered and described. The first reports on representatives of the phylum were associated with extreme thermophilic environments, e.g., the hot springs mentioned above [50] and hydrothermal vents [51]. Further studies provided growing evidence that representatives of the phylum can occupy other environmental niches as well. Interestingly, their high contribution (reaching as much as 60% of the total community) has been confirmed in permafrost [52,53], which denies their exclusively thermophilic character. Additionally, a negative effect of the increased temperature has been observed in this specific location [52]. *Caldiserica* was also identified in lake waters both in deep anoxic parts [54] and, surprisingly, the upper layers [55]. The results presented in this study pointing to the presence of these bacteria in the sediments of the shallow subsidence reservoir support the recent discoveries of *Caldiserica* capability to live in mesophilic conditions or even temperatures close to zero (such as those occurring in shallow lake sediments in winter) and to cope with oxidative stress. Bearing in mind the variety of environments occupied by *Caldiserica* (evidence of genomic diversity), it may be expected that the methodological progress in the field of environmental genomics will soon facilitate description of other species belonging to this phylum colonising various environments and eluding culturing attempts. 

Other representatives of microbial “dark matter” that have recently come into the limelight and were found in the Szczecin reservoir sediments were those of *Methanomassiliicoccus*. This methanogenic methylotrophic *Archaea* was first described in human faeces [56]. Further, its relatives were found in the intestinal tracts of other organisms [57] or faeces-affected sludges (e.g., from wastewater treatment plants) [58,59]. Most research of these microorganisms focuses on their interaction with human health [60,61,62] or the unique methylotrophic but H_2_-dependent metabolism [63]. *Methanogenic Thermoplasmata* (including *Methanomassilicoccales*) use a reduced methanogenic pathway, in which methanol and other methylated compounds are reduced to methane in the presence of H_2_ [56]. This metabolic pathway has long been considered to have minor environmental importance, as it was reported to be used by only two methanogenic species. Recently, the visibility of *Thermoplasmata*-related sequences has been enhanced by the description of culturable species and *M. luminensis* genome sequence deposition in public databases [64]. The ecology of this newly described archaeal phylum is currently being recognized. Comparative phylogenetic studies performed by Paul et al. [65] have implied that *Methanomassiliicoccales* may be a part of the microbiome occurring in various environments. To date, these assumptions have been confirmed for extreme environments such as hot springs [66], formation waters connected with oil reservoirs [67], wetland soils [68], lake sediments [69], and deep subsurface (coal) [70]. The present study, indicating that the Szczecin reservoir sediment (SZN) is occupied by *Methanomassiliicoccaceae*, is in line with the aforementioned discoveries and is the second report, after Fan and Xing [69], on their presence in lake sediments. Surprisingly, the enrichment cultures in the presented experiment revealed that this group of methanogens is especially enriched in the presence of acetate. All currently published enrichment cultures and a sole *M. luminyensis* isolate were obtained on methanol or methylamines as a carbon source and H_2_ [68]. Hence, it could be expected that *Methanomassiliicoccus* would find the best growth conditions in medium H(+)CO_2_/H_2_, where organic substrates and hydrogen were added. Surprisingly, only 0.17% of the sequences in this treatment were affiliated to this genus, vs. nearly 2.5% in H(+)acet, which means that it was 27 times more abundant in these conditions than in the original sediment. These results contrast with previous studies of *Methanomassiliicoccus*-containing enrichment cultures. In experiments presented by Lv et al. [67], addition of acetate to the culture medium resulted in replacement of *Methanomassiliicococcacae* by *Methanosaeta* and *Methanosarcina* (both known for acetotrophic metabolism). In the present study, *Methanosarcina* was also dominant in all acetate-amended cultures (which is not a surprise) but in H(+)acet *Methanomassiliicoccus* accounted for nearly 12% of all methanogens. The difference between these two experiments may result from the different origin of the inoculates. Lv and co-workers [67] investigated communities derived from oil production waters, while our study was developed based on a community retrieved from a shallow lake sediment; therefore, they may represent distinct species. The utilization of acetate by *Methanomassiliicoccaceae* in lake sediments may be a result of adaptation to in situ conditions. Shallow lake littoral zones are often overgrown by aquatic macrophytes. The roots of these plants are known to exude organic acids. Acetate is thus an abundant substrate in the sediment and, provided anaerobiosis is maintained, can be used by methanogens. Our hypothesis pointing to stimulation of *Methanomassiliicoccaceae* by acetate exudates seems to be confirmed also by Fan and Xing [69], who reported that representatives of the genus are more abundant in littoral- macrophyte overgrown sediments than in deeper parts of the lake (dominated by algae). Also, the sequence retrieved from the SZN metagenome exhibited high similarity with 16SrDNA fragments isolated from environmental littoral samples overgrown by reed (AB896665.1) and rice (KU522088.1; GU134476.1).

## 4. Materials and Methods

The research material was bottom sediment (0–5 cm) of an endorheic reservoir named Szczecin (51°20’18″N 22°59’45″E, Lublin region, Poland). The reservoir developed in 1995 in the post-mining subsidence as result of underground operations carried out in the nearby “Bogdanka” coal mine (Lublin Coal Basin). Its area is about 100 ha and the maximum depth is approximately 2.5 m [71]. The reservoir basement is formed of sandy-clay soil, which is characteristic of the area [72]. The water in the reservoir has become eutrophic since nutrients are likely to get into the water from nearby fields [73]. Sediments were taken in triplicate in the summer of 2016 (Piston sampler, Eijkelkamp, Nederland). They were transferred into tightly closed sterile jars, transported to the laboratory, and kept at 5 °C for a few days until the experiment started and at −20 °C for the molecular analyses. 

### 4.1. Physical and Chemical Analysis

The sediment moisture was determined gravimetrically by oven-drying to a constant weight at 105 °C, immediately after collecting the samples. Reaction (pH) and redox potential (Eh) were determined using a multifunctional potential meter Sension+ MM150 and a multi-sensor for pH and Eh (HACH, USA). The carbon content in dry samples was determined by means of TOC-V_CSH_ with an SSM-5000A module autoanalyzer (Shimadzu, Japan). The total organic carbon (TOC) amount was calculated from the difference between total carbon (TC) and inorganic carbon (IC) [74]. Bioavailable forms of nitrogen (nitrite, nitrate, ammonium) and phosphorus were measured in bottom sediment extracts (sediments with deionized water and with 0.5 M NaHCO_3_ for nitrogen and phosphorus forms respectively) using an AA3 autoanalyzer (Braun & Luebbe, Germany) after filtering through filter paper (Munktell, grade 390, Germany) according to the method described by Banach [75].

### 4.2. Incubation Experiments

#### 4.2.1. Assessment of the Methane Production Rate in Sediment

Each stage of methanogenic incubation was prepared aseptically in anoxic conditions with a nitrogen atmosphere (glove box chamber, Labconco, Kansas City, MO, USA). At the beginning, the natural capability of methane production in the sediments (SZN) was tested. Ten millilitres of bottom sediments were placed in 60-mL serum vials, closed with butyl rubber septa, and capped. Samples were incubated at temperatures of 10, 20, 30, and 40 °C for up to 90 days, always in triplicate. 

#### 4.2.2. Cultivation Conditions of the Enrichment Cultures

The media were prepared according to Horn [76] with slight modifications. Sterile (autoclaved) medium containing (milligrams per litre) (NH_4_)_2_SO_4_—25; CaCl_2_ ·x 2H_2_O—10; MgCl_2_ x 6H_2_O—5; NaCl—200; NH_4_Cl—200; and KH_2_PO_4_—200, supplemented with a vitamin solution (10 mL) and a trace element solution (10 mL), was used as a basic mineral solution (H(−)) [77]. The medium was prepared without addition of cysteine or other reducing agents, to facilitate assessment of the natural reduction potential of the investigated microbial communities. The hermeticity of the bottles (oxygen intrusion) was monitored chromatographically. No resazurin was added. The pH of the medium was adjusted to 7 with KOH. The experimental treatments were prepared with tryptone, yeast extract, acetate, and CO_2_/H_2_ (20:80 *v*/*v*) as shown in Table 5. The initial headspace pressure in N_2_-filled containers was close to atmospheric. In treatments with CO_2_/H_2_, nitrogen was replaced with a mixture of the given gases with overpressure of approximately 150 kPa [78]. 

The enrichments were carried out in 120 mL serum vials in a 1:50 proportion of the inoculating material to the fresh medium in the dark and without shaking for up to 90 days. The bottom sediments were used as an inoculum in the first stage of the enrichment (S1), whilst the liquid enrichment from the previous stages (S1 and S2, respectively) were used in the second (S2) and third (S3) stages. Incubations of the SZN and S1 enrichment cultures were carried out at temperatures between 10 and 40 °C. The number of temperature variants at subsequent stages was reduced by those characterized by the lowest methanogenic activities.

The optical density of the enrichment (OD_660_) was measured using a UV-1800 (Shimadzu, Japan) spectrophotometer [79].

#### 4.2.3. Chromatographic Analyses and Calculation of the Methane Production

The rate of CH_4_ production and, also loss of CO_2_ and H_2_ in some research enrichment variants, was determined using a gas chromatograph (GC 3800, Varian, USA) equipped with flame ionization (FID, 200 °C) and thermal conductivity (TCD, 120 °C) detectors in series and with the use of two types of columns: a Poraplot Q 0.53 mm ID (25 m) and a Molecular Sieve 5A 0.53 mm ID (30 m) connected together, were used with helium as the carrier gas [74,80]. Methane production rate (MP) was determined on the basis of the linear increase in the methane concentration in time and expressed as a mg of produced CH_4_ per litre of bottom sediments or enrichment medium per day (mg CH_4_ L^−1^ d^−1^).

### 4.3. DNA Extraction and NGS Procedure

Microbial genomic DNA from the bottom sediment and cultures was extracted using a PowerLyzer PowerSoil DNA Isolation Kit (Quiagen, Hilden, Germany) according to the manufacturer’s instructions. 0.25 g of fresh material from the bottom sediments and cell pellets harvested from 4 mL of the enrichments (after centrifugation, 13000× RPM, 5 min) in the last incubation step (S3) were used for isolation. The presence of DNA was confirmed by electrophoresis in a 1% agarose gel with 1×TBE buffer and a SimplySafe™ (EURX) stain for detection of nucleic acid. The V3–V4 region of 16S rRNA gene amplicons were sequenced with the MiSeq Illumina technology (Genomed Inc, Poland).

### 4.4. Bioinformatic Analysis

Amplicon sequence variants (ASVs) were resolved with DADA2 version 1.8 package [81] in R version 3.5.1 [82]. Based on the sequence quality plots, forward and reverse reads were trimmed, respectively, to 250 and 240 bp, and primer sequences were removed from all reads. The following filtering parameters were used: maxN = 0, maxEE for the forwards reads = 3 and for the reverse reads = 5, truncQ = 2. Other parameters were set to default. The error rates were estimated by learnErrors using one million reads. Sequences were dereplicated using derepFastq with default parameters and exact sequence variants were resolved using *dada*. Next, removeBimeraDenovo was used to remove chimeric sequences.

Taxonomy was assigned against the latest version of the RDP database (11 version) using a Naïve Bayesian Classifier [83] with the minboot parameter set to 80. The resulting taxonomy and read-count tables constructed in DADA2 were appropriately converted and imported into the phyloseq (1.22.3) package [84]. Sequences identified as chloroplast and mitochondria were removed.

16S rRNA amplicon sequencing data generated in this study were deposited in the NCBI Sequence Read Archive (SRA) under the BioProject number PRJNA514232.

### 4.5. Metabolic Pathways Prediction Using PICRUSt Software

Using PICRUSt (Phylogenetic Investigation of Communities by Reconstruction of Unobserved States, software) [85], the functional genes were reconstructed based on bacterial 16S rRNA gene sequences. The input ASVs from the dada2 workflow were clustered into OTUs (operational taxonomic units) using the cluster-features-closed-reference algorithm implemented in qiime2 software [86] with the 97% similarity threshold and using the GreenGenes (13.8) database as a reference. The number of 16S rRNA gene copies was normalized using the normalize_by_copy_number.py algorithm implemented in PICRUSt software. Metagenomes were predicted using the predict_metagenomes.py algorithm, and the functional genes were annotated in the KEGG database using the categorize_by_function.py algorithm.

## 5. Conclusions

The results obtained in this study show that our current understanding of microbial processes leading to degradation of organic matter to methane is still far from complete. We have shown that in addition to the already identified relations in the microbial world leading to methane production, there is an enormous scope of biotechnological potential hidden in the microbial “dark matter”. In light of the great diversity and hardly graspable dynamics of microbial communities in the natural environment, our knowledge is still insufficient, although it is expanding year on year. In the present work, we have shown that the enrichment cultures diversified by the available carbon substrate may serve as a useful tool for the study of microbial ecology and physiology, giving a chance to highlight the activity of microorganisms (e.g., *Caldisericum*, *Methanomassiliicoccus*, *Methanothrix*) that escape recognition using other methods (e.g., based on pure culture analysis). Biotechnology can gain from studies of interspecies relations. Our research suggest that *Caldisericum* ssp., which are likely to cooperate with methanogens, may have an unexploited potential for the biogas production industry.

Furthermore, their high methane production rate and self-reduction capacity imply that further studies should be undertaken to test the potential of the cultures isolated from lake sediments for biogasification of various organic material. Furthermore, we suggested that more efforts should be made to investigate the role of *Caldiserica* in methane formation processes due to its putative positive impact that may have biotechnological application.

## Figures and Tables

**Table 1:** Characteristics of investigated sediments (± SD).

N-NO_2_	N-NO_3_	N-NH_4_	P-PO_4_	TC	IC	TOC	Moisture	pH	Eh
mg kg d.w.^−1^				g kg d.w.^−1^			%		mV
0.11 ± 0.01	0.30 ± 0.04	28.21 ± 4.88	10.05 ± 0.25	48.0 ± 0.8	0.00	48.0 ± 0.8	58.71 ± 2.28	5.46 ± 0.02	−265.63 ± 2.89

**Table 2:** Lag phase duration in particular media and stages of enrichment.

Stages of Bacterial Cultivation	Temperature (°C)	Lag Phase (d)				
		H(−)CO_2_/H_2_	H(−)acet	H(+)	H(+)CO_2_/H_2_	H(+)acet
S1	10	14	31	14	14	31
	20	10	21	10	10	10
	30	1	10	1	1	1
	40	1	7	1	1	1
S2	20	6	6	6	1	6
	30	1	7	1	1	1
	40	1	6	1	1	1
S3	20	-	4	-	-	-
	30	1	-	1	1	1

**Table 3:** Changes in physicochemical parameters between the start and the end of the particular stages of incubation (± SD).

Stages of Bacterial Cultivation/Parameters		Medium Variants				
		H(−)CO_2_/H_2_	H(−)acet	H(+)	H(+)CO_2_/H_2_	H(+)acet
		Δ (start to end)				
S1	pH	−0.5 ± 0.01	−0.24 ± 0.03	−0.13 ± 0.02	−0.21 ± 0.00	−0.14 ± 0.01
	Eh (mV)	−383.20 ± 0.32	−264.44 ± 0.06	−337.27 ± 0.40	−368.20 ± 0.10	−303.87 ± 0.25
	OD	na	na	na	na	na
S2	pH	−0.24 ± 0.01	1.07 ± 0.00	0.37 ± 0.00	−0.36 ± 0.01	0.79 ± 0.02
	Eh (mV)	−330.60 ± 0.10	−463.17 ± 0.30	−347.77 ± 0.49	−358.20 ± 0.10	−467.53 ± 0.84
	OD	0.09 ± 0.02	0.08 ± 0.02	0.15 ± 0.01	0.09 ± 0.02	0.14 ± 0.01
S3	pH	−1.18 ± 0.02	1.49 ± 0.00	0.16 ± 0.04	−1.56 ± 0.04	0.76 ± 0.01
	Eh (mV)	−405.03 ± 0.49	−466.33 ± 0.15	−400.50 ± 1.42	−465.80 ± 0.21	−433.16 ± 1.05
	OD	0.09 ± 0.01	0.07 ± 0.02	0.16 ± 0.01	0.16 ± 0.02	0.16 ± 0.01

**Table 4:** Biodiversity indices of the experimental treatments.

Treatment	Stage	Observed	Chao1	ACE	Shannon	Simpson	InvSimpson	Fisher
SZN	-	2536	2585.96	2565.61	7.06	1.00	422.83	559.06
H(−)CO_2_/H_2_	S1	1227	1232.69	1229.26	5.45	0.98	48.19	207.52
	S2	230	230.00	230.00	3.25	0.91	11.27	28.11
	S3	96	96.00	96.00	1.40	0.53	2.11	10.55
H(−)acet	S1	1206	1206.56	1206.80	5.41	0.98	64.49	202.84
	S2	317	317.00	317.14	3.64	0.94	15.39	41.54
	S3	184	184.00	184.20	2.59	0.80	5.00	22.77
H(+)	S1	996	996.30	996.43	5.10	0.97	33.28	158.44
	S2	353	353.00	353.14	4.07	0.96	25.17	48.10
	S3	238	238.00	238.00	3.38	0.92	11.96	30.61
H(+)CO_2_/H_2_	S1	574	574.00	574.14	4.93	0.98	66.40	85.08
	S2	121	121.00	121.00	1.89	0.69	3.27	13.60
	S3	97	97.00	97.00	2.67	0.87	7.63	10.80
H(+)acet	S1	530	530.00	530.00	4.72	0.97	39.57	69.79
	S2	335	335.00	335.14	3.54	0.91	10.83	44.04
	S3	241	241.00	241.28	3.83	0.95	20.53	31.56

**Table 5:** Variants of the media.

Media code	Tryptone (g L^−1^)	Yeast Extract (g L^−1^)	CH_3_COONa (g L^−1^)	Under Gases
SZN	Natural sediment with no additives			N_2_
H(−)CO_2_/H_2_	0	0	0	CO_2_/H_2_ (20:80 *v*/*v*)
H(−)acet	0	0	2	N_2_
H(+)	0.5	0.5	0	N_2_
H(+)CO_2_/H_2_	0.5	0.5	0	CO_2_/H_2_ (20:80 *v*/*v*)
H(+)acet	0.5	0.5	2	N_2_

H(−)—basic mineral solution, H(+)—basic mineral solution supplemented with tryptone and yeast extract.

## Abbreviations

KEGG	Kyoto Encyclopedia of Genes and Genomes
PICRUSt	Phylogenetic Investigation of Communities by Reconstruction of Unobserved States, software
